# 

**Published:** 1919-02

**Authors:** 


### Old Jokes That We Have Met.

AN IMPORTANT POINT—"Your wife has imaginary ailments."

"Um."

"I'll just give her some imaginary medicine."

"Um. What kind of a bill are you going to render in this case, doc?"

---

CRUEL DECEPTION—"How do you manage to keep your cook so long?"

"My husband has promised her that if she is working for us when we strike oil he will buy her an auto."

"But I did not know you had any oil prospects?"

"We haven't."—Houston Post.

---

"OH, DEAR," said a little girl struggling with her artihmetic, "I wish I were a rabbit in Australia."

"Why?" asked the mother.

"Because teacher says rabbits multiply so rapidly."

---

*Shrewd Dentist.*

RANKIN—"Isn't that Dr. Diggendelve, the wealthy dentist?

PHYLE—"Yes."

RANKIN—"I wonder why he is eating his meals in this restaurant?"

PHYLE—"He always patronizes the restaurants during the blackberry pie season, so he can pick out the people whose teeth need filling."—Judge.

---

"One dollar, please," said the dentist. "A dollar! But your sign reads 'Painless extraction of teeth free.' "

"Just so. But as you hollered a bit, this does not apply to your case. I do my painless extracting free, exactly as I claim. When it hurts, I charge for it. One dollar, please."

---

COLLIE—"When I was a boy you know, the doctor said if I didn't stop smoking cigarettes I would become feeble minded."

BESSIE—"And why didn't you stop?"

